# Monitoring of risk perceptions and correlates of precautionary behaviour related to human avian influenza during 2006 - 2007 in the Netherlands: results of seven consecutive surveys

**DOI:** 10.1186/1471-2334-10-114

**Published:** 2010-05-12

**Authors:** Onno de Zwart, Irene K Veldhuijzen, Jan Hendrik Richardus, Johannes Brug

**Affiliations:** 1 Division of Infectious Disease Control, Municipal Public Health Service Rotterdam-Rijnmond, Rotterdam, the Netherlands; 2 Department of Public Health, Erasmus MC, University Medical Center Rotterdam, Rotterdam, the Netherlands; 3 The EMGO-Institute for Health and Care Research, VU University Medical Center, Amsterdam, the Netherlands

## Abstract

**Background:**

Avian influenza (AI) is a public health challenge because of ongoing spread and pandemic potential. Non-pharmaceutical measures are important to prevent the spread of AI and to contain a pandemic. The effectiveness of such measures is largely dependent on the behaviour of the population. Risk perception is a central element in changing behaviour. This study aimed to investigate perceived vulnerability, severity and precautionary behaviour related to AI in the Netherlands during seven consecutive surveys in 2006 - 2007 as well as possible trends in risk perception and self-reported precautionary behaviours.

**Methods:**

Seven web-based surveys were conducted including 3,840 respondents over a one-year period. Time trends were analyzed with linear regression analyses. Multivariate analysis was used to study determinants of precautionary behaviour.

**Results:**

While infection with AI was considered a very severe health problem with mean score of 4.57 (scale 1 - 5); perceived vulnerability was much lower, with a mean score of 1.69. While perceived severity remained high, perceived vulnerability decreased slightly during a one-year period covering part of 2006 and 2007. Almost half of the respondents (46%) reported taking one or more preventive measures, with 36% reporting to have stayed away from (wild) birds or poultry. In multivariate logistic regression analysis the following factors were significantly associated with taking preventive measures: time of the survey, higher age, lower level of education, non-Dutch ethnicity, vaccinated against influenza, higher perceived severity, higher perceived vulnerability, higher self efficacy, lower level of knowledge, more information about AI, and thinking more about AI. Self efficacy was a stronger predictor of precautionary behaviour for those who never or seldom think about AI (OR 2.3, 95% CI 1.9 - 2.7), compared to those who think about AI more often (OR 1.5, 95% CI 1.2 - 1.9).

**Conclusions:**

The fact that perceived severity of AI appears to be high and remains so over time offers a good point of departure for more specific risk communications to promote precautionary actions. Such communications should aim at improving knowledge about the disease and preventive actions, and focus on perceived personal vulnerability and self efficacy in taking preventive measures.

## Background

Infectious diseases are once again among the major public health challenges. The SARS epidemic of 2003 showed not only that there are new unknown viruses which can have severe health consequences, but also made clear how fast a disease can spread globally, what the societal and economic impact can be, as well as how the media may contribute to awareness and public concerns [1,2]. While SARS came as a surprise, since April 2009 the world is confronted with a new influenza H1N1 pandemic.

Up to July 1, 2009 the World Health Organization (WHO) has confirmed 436 human cases of avian influenza (AI) in 15 countries, mostly in South-East Asia, with 262 fatalities [3]. Outbreaks of AI among (wild) birds or poultry have been reported in 61 countries [4]. So far, human AI infections were limited to people who had been in close contact with (wild) birds or poultry. Nonetheless, the possibility of an adaptation of the current H5N1 AI virus might lead to a new influenza virus which would be easily transmitted from human-to-human and may thus lead to a new influenza pandemic. After the three influenza pandemics in the 20^th ^century, the 'Spanish influenza' in 1918, the 'Asian influenza' in 1957 and the 'Hong Kong influenza' in 1968 the world is now witnessing a new influenza pandemic caused by the new H1N1, which has caused 134,503 cases with 816 deaths up to July 27, 2009 [5]. In June 2009, WHO increased the phase of pandemic alert to 6, indicating that a global pandemic is under way [6].

In preparing for an influenza pandemic the development of vaccines as well as the stockpiling of antiviral drugs has received most attention. While developments have been made and some countries have now a (limited) amount of antiviral drugs, some argue that most countries fail the precautionary principle because they have not ensured enough effective drugs [7]. Furthermore, focusing on vaccines and antiviral drugs only will probably not be enough to limit the consequences of an influenza pandemic, as these will likely neither be available in time nor in the right quantities, and non-medical interventions will be of great importance in the control of the epidemic [8]. The WHO has proposed a number of measures in case of an influenza pandemic: recommendations on personal hygiene, quarantine, travel restrictions, closure of schools and other public gatherings [9,10]. A historical analysis by Markel and colleagues of the reaction to the 'Spanish influenza' in several cities in the United States showed that cities which took earlier nonpharmaceutical interventions, such as school closures, public gathering bans and isolation and quarantine, and sustained these measures, had greater delays in reaching peak mortality, lower peak mortality rates and lower total mortality [11]. During the SARS outbreak the importance of nonpharmaceutical interventions was also shown; quarantine and hygiene measures helped the control of the SARS outbreak [12].

The effectiveness of both pharmaceutical and nonpharmaceutical interventions is largely dependent on the behaviour of the population, i.e. compliance with the recommended preventive measures. For promotion of adequate precautionary behaviour among the populations, public health authorities need to know how people perceive risks, how they perceive the effectiveness and acceptance of nonpharmaceutical interventions and whether they will trust and be willing and able to use the information from public health and other authorities. Based upon earlier outbreaks of infectious diseases, there is only very limited information on these issues. In 2003 there was a severe outbreak of H7N7 AI in the Netherlands, resulting in one fatality [13]. For poultry workers, poultry farmers and their families, specific measures were advised such as the use of facial masks and goggles to prevent infection, and taking anti-viral therapy. Adherence to these measures was low even if people were directly at risk for infection [14].

Risk perception is a central construct in various behaviour theories [15]. Risk perception has been studied intensively in relation to environmental and technical-industrial risk. Smith distinguishes two approaches in the field of risk perception studies. The first is the so-called 'realistic' approach focusing on measuring the objective risk of a specific threat or danger, which can be measured independently from the social context [1,16,17]. Much of the early work of Slovic, which included comparisons of perceptions of risk, can be placed in this tradition [18-20]. The second approach is the 'social constructionist' approach, where the perception of risk is the result of social and cultural processes and is shaped by these processes (see e.g. the works of Joffe [21] and Beck [22]). In the present study into risk perception of AI we combine a realistic approach - since there is a real risk for human AI and the possibility of influenza pandemic and specific knowledge about these - with a social constructionist approach focusing on how people perceive risks and what actions they take.

Based upon Protection Motivation Theory we distinguish between the perceived severity of a disease, described by Brewer et al. as the extent of harm a hazard would cause, and the perceived vulnerability (often also described as perceived likelihood or risk perception as such), described as the probability that one will be harmed by the hazard [15,23,24]. Additionally, comparative vulnerability can be defined as the probability that one will be harmed by the hazard compared to others of the same age and gender. Risk perceptions are often biased. A low comparative vulnerability, which may indicate unrealistic optimism, is regularly observed towards familiar risks that are perceived to be largely under volitional control. In this optimistic bias context, people perceive their comparative vulnerability compared to others of the same sex and age as lower. The opposite, when people perceive their comparative vulnerability to be higher than others of the same sex and age, may indicate a pessimistic bias, which is more likely for new risks that are perceived as uncontrollable. The latter might be the case with new emerging infectious diseases, like AI or an influenza pandemic [18,25-27].

Protection Motivation Theory suggests that, apart from risk perception, response efficacy and self efficacy are two key determinants of precautionary behaviour. Response efficacy relates to the belief of people in the effectiveness of the available protective actions, for example hygienic measures. Self efficacy relates to a person's perception of their ability to engage in such protective actions, e.g. that they are able to carry out the proposed hygienic measures. Several reviews and meta-analyses focusing on the effects of fear, risk perception and fear appeals on health behaviours have suggested that higher risk perception will only predict precautionary behaviour when people believe that effective protective actions are available (in case of sufficient response efficacy) and when they have confidence that they have the opportunities and abilities to engage in such protective actions (sufficient self-efficacy) [28,29].

While risk perception is an important factor in many health psychology models, there is ongoing discussion about the (magnitude of the) effect of risk perception on precautionary behaviours [15]. While associations between risk perceptions and precautionary actions have been found, they are often small. In their recent meta analysis Brewer and colleagues showed that perceived likelihood, susceptibility and severity were all significantly associated with whether people got vaccinated against influenza with the largest effect for likelihood (pooled r = 0.26). Since most studies into risk perception of infectious diseases do not take place during outbreaks of infectious diseases, it is difficult to include measures of precautionary behaviour in such research. Most of such studies therefore focus on future behaviour or intended behaviour in case of an outbreak [30,31]. In the present study we were able to include self-reported precautionary behaviour related to AI at the time of the data collection.

Since the SARS outbreak research into risk perception of infectious diseases has gained interest. Several studies that were conducted during the SARS outbreak and its aftermath, indicated a relatively high level of risk perceptions in the United States, and relatively lower levels in Hong Kong and the Netherlands [32-34]. Because these studies were mostly single cross-sectional surveys, it is to date unknown how risk perceptions evolve over time and are affected by such issues as news coverage in the popular media or other events drawing attention to a possible outbreak. Only a few studies have studied risk perception related to emerging infectious diseases over a longer period of time [35,34].

The present study explored risk perception, efficacy beliefs and precautionary behaviour related to human AI in seven consecutive surveys in the Netherlands. As far as we know our study is the first with such a large sample and consecutive surveys looking into actual self-reported precautionary behaviour instead of reports on would-be behaviours in case of an outbreak or other event. To explore if risk perceptions were specific for human AI, risk perception related to other (infectious) diseases were also investigated. The study had the following specific objectives:

- To study levels of perceived severity, perceived vulnerability and comparative vulnerability of infection with human avian influenza and compare these with perceptions related to other diseases and conditions such as common cold, diabetes, HIV, high blood pressure, tuberculosis, food poisoning and a heart attack.

- To explore knowledge of avian influenza, the amount of information people had received about avian influenza and how often they thought about it.

- To analyse correlates of risk perception of avian influenza, and gender, age, having children below the age of twelve, ethnicity, education, thinking about AI, knowledge about AI, the amount of information received and being vaccinated against influenza.

- To study precautionary behaviour related to avian influenza and its potential determinants.

- To analyse possible trends in risk perception and self-reported precautionary behaviours over a period with changing risks and publicity related to possible outbreaks.

## Methods

Seven web-based surveys among random samples from an Internet panel were conducted. At the time of data collection, the panel consisted of approximately 15.000 members of whom the distribution of demographic variables (gender, age, region, and level of education) was comparable to the Dutch adult population at large. For each survey an independent random sample was drawn of between 700 and 952 panel members from 18 years and older. These panel members received an invitation to participate by email. Each survey was online between 8 and 13 days. Panel members received 1.50 Euro in credits for completion of a survey. When panel members have a certain amount of credits for participating in surveys they can exchange their credits for a gift cheque covering the exact amount of credit. For survey studies under the Dutch Medical Research Involving Human Subjects Act approval of an appropriate ethics committee is not required.

Five of the surveys were periodical with three month intervals. The two remaining surveys were conducted immediately after two relevant events. The first event, in August 2006, was a suspected infection of two owls with AI in the Rotterdam zoo. The survey in August 2006 took place before the Department of Agriculture issued a press release informing that no H5N1 infection had been diagnosed in the owls. The second event was news coverage of an outbreak of AI at a turkey farm in the UK, which led to new requirements to keep birds under cover in the Netherlands in February 2007. During the full 13 month period of serial surveys neither human cases nor infections among birds or poultry were observed in the Netherlands.

In line with Protection Motivation Theory we developed a survey that focused on risk perception, precautionary behaviour, and self and response efficacy. The survey was based on an earlier survey used for studies into risk perception and pandemic influenza [33]. As we used an existing internet panel with known data on gender, age, country of birth and level of education, questions on these demographics were not included in the questionnaire. The questionnaire started with additional demographic questions concerning the country of birth of both parents, size of the household and whether there were children younger than 12 years in the household. Next, a number of questions were asked about perceived severity and susceptibility on five point answering scales. First it was asked how serious it would be for the respondent to get one of the following diseases and conditions in the next year: diabetes, a regular cold, hiv/aids, high blood pressure, AI, tuberculosis, a heart attack and food poisoning. Answer possibilities ranged from very serious to not serious at all. The next question included the same diseases and condition and asked how likely it would be that the respondents themselves would get the disease in the coming years (very small chance - very large chance). These formulations are in line with the conditioned risk questions as discussed by Brewer and colleagues in their recent meta-analysis of the relation between risk perception and influenza vaccination [15]. To assess comparative vulnerability respondents were asked whether compared to someone of the same age and gender in the Netherlands they would have a smaller or larger chance to get one of the diseases and conditions in the coming years (a much smaller chance - a much larger chance).

The next question asked how often people thought about AI (never - always). Self efficacy was assessed by asking 'How sure are you that you yourself can prevent getting AI when AI reaches the Netherlands' (not sure, - very sure). Respondents were also asked whether they had taken measures to prevent themselves getting infected with AI. Possible measures included not getting in touch with (wild) birds or poultry, not going to areas with AI, paying more attention to hygiene, eating less or no chicken or poultry, cancelled or didn't book a holiday to an area with AI, getting oneself vaccinated against influenza, avoiding shaking hands, keeping the cat indoors, avoid gatherings of people, buying antiviral drugs, buying a mouth mask, something else or done nothing. We categorized these measures as measures recommended by health authorities (not getting in touch with wild birds or poultry), non-effective measures (eating less or no chicken or poultry, getting oneself vaccinated against influenza) and measures which were not recommended although they may have some preventive effect (all the other measures). The Dutch questionnaire is available on http://www.ggd.rotterdam.nl/Rotterdam/Openbaar/Diensten/GGD/Pdf/IZB/Vragenlijst%20Vogelgriep%20Onderzoek%20extra%20 meting%20aug06.pdf.

We also asked how much information people had received about AI (ranging from nothing to very much), and assessed knowledge of AI based upon four questions. First of all 'The avian influenza virus can be transmitted from human to human' (false). Secondly 'There is a vaccine that protects humans against infection with the avian influenza virus' (false). Thirdly 'In the Netherlands in 2006 people have died as a result of an infection with the avian influenza virus' (false) and finally 'By eating chicken or poultry someone can become infected with the avian influenza virus' (false). The right answers were those answers that were in line with the public information in the Netherlands at the time of the surveys. The questionnaire included a question on whether the respondent was vaccinated against influenza in the last year and whether he or she kept chicken or poultry.

Mean scores and 95% confidence intervals were calculated for the perceived severity, vulnerability and comparative vulnerability of several diseases and conditions. Differences in background characteristics (gender and level of education) between the surveys were explored with Chi-square tests. Level of education was divided in three categories: lower education (primary school, lower general secondary school, lower vocational school, or less), intermediate level education (high school or medium level vocational school), or higher education (university or college degree). Differences in age and mean scores between surveys were tested pair wise, taking multiple testing into account, with the Bonferroni post-hoc test in Univariate Analysis of Variance (ANOVA). Time trends were analyzed with linear regression analyses with perceived severity, perceived vulnerability, amount of information, knowledge and the different preventive measures as dependent variables and time (the survey) as the main independent variable and gender, age and level of education as covariates.

Univariate associations of several determinants with perceived vulnerability were tested with ANOVA or T-test. To study correlates of precautionary behaviour, a new dichotomous variable 'precautionary measures' was defined and coded 1 (yes), if respondents had taken any of the specific measures, and coded 0 (no) if respondents had done nothing. Univariate logistic regression analyses were performed, with self reported characteristics as independent variables and taking precautionary measures as the dependent variable. For the odds ratios, 95% confidence intervals (CI) were calculated. Variables showing an association of p < 0.1 were included in the multivariate analysis. Variables were included blockwise, with the time of the survey and general characteristics in the first step, severity and vulnerability were added in the second step, self efficacy in the third step, and knowledge, amount of information received and thinking about AI in the fourth step. All first order interactions between the variables added after the first step were assessed. The final model was run after excluding variables with an association of p > 0.1.

## Results

In total 3,840 respondents participated in the seven surveys; per survey the number of participants varied between 467 and 650. Overall response was 64% and varied between 55% and 77% (see Table 1). In all but one, (i.e. the August 2006 survey) the majority of respondents were women, in total 54%. Compared to the general Dutch population, where 51% is female, women are slightly overrepresented in our surveys. The proportion of women in the first 4 surveys was lower compared to the last 3 surveys (51% vs. 58%, χ^2 ^(df) = 16.5 [1]; p < 0.001) (Table 1). Mean age was 45 years (range 18-86, SD 14.8), with respondents in survey 1 being significantly younger (41.3) than respondents in the other surveys (46.0), (F(df) = 51.0[1]; p < 0.001). Compared to the general Dutch population, people aged 45-59 years were somewhat overrepresented in the survey (31% versus 27%), and people aged 60 years and older underrepresented (20% versus 24%). Over all surveys, 26%, 39% and 35% of respondents had received lower, intermediate and higher education respectively. This distribution is 27%, 44%, and 29% in the general population, which shows overrepresentation of participants with high educational level and underrepresentation of those with intermediate educational level. 11% of the participants were of non-Dutch origin and 23% had been vaccinated against influenza. Children under 12 years lived in 22% of the households. Only 4% kept chicken or poultry at home.

**Table 1:** Participation rates and distribution of general characteristics in the study population.

	*N*	*%*
Invited	5995	
Participants, response	3841	64%
Gender		
Male	1765	46%
Female	2046	54%
Age (mean)	45.3	
Education*		
Low	1000	26%
Intermediate	1479	39%
High	1332	35%
Children <12 in household		
Yes	845	22%
No	2966	78%
Ethnicity		
Dutch	3398	89%
Non-Dutch	413	11%
Vaccinated against influenza		
Yes	887	23%
No	2909	76%
Keeping chicken or poultry		
Yes	137	4%
No	3674	96%

* Educational level is defined as: low: primary school, lower general secondary school, lower vocational school, or less; intermediate: high school or medium level vocational school; high: college or university degree.

Infection with AI was perceived as a (very) severe health problem by 91.8% of the study population with a mean score of 4.57 (95% CI 4.55 - 4.60; scale 1 - 5); 0.7% reported (very) high perceived vulnerability, mean = 1.69 (95% CI 1.66 - 1.71, scale 1 - 5) (see Table 2). Comparative vulnerability for AI was 2.59 (95% CI 2.56 - 2.61, scale 1 - 5, whereby 3 stands for an equal chance to others of the same age and gender). Compared with other diseases, getting infected with AI was perceived as very serious with a score of 4.57 on a scale from 0-5 (see Table 2). Only HIV, 4.92 (95% CI 4.91 - 4.93), and heart disease, 4.82 (95% CI 4.80 - 4.83), had a significantly higher perceived severity. Perceived severity of other diseases and conditions varied from 4.18 (95% CI 4.16 - 4.21) to 1.87 (95% CI 1.85 - 1.89) for a common cold. In contrast perceived vulnerability for common cold was the highest with 3.64 (95% CI 3.61 - 3.67), while for HIV it was the lowest at 1.23 (95% CI 1.21 - 1.24). Comparative vulnerability of HIV was also low at 1.88 (95% CI 1.85 - 1.91), while for a common cold it was 3.07 (95% CI 3.05 - 3.09).

**Table 2:** Perceived severity, vulnerability and comparative vulnerability (mean and 95% CI).

	*Severity*	*Vulnerability*	*Comparative vulnerability*
	Mean (95% CI)	Mean (95% CI)	Mean (95% CI)
HIV	4.92 (4.91 - 4.93)	1.23 (1.21 - 1.24)	1.88 (1.85 - 1.91)
Heart attack	4.82 (4.80 - 4.83)	2.26 (2.23 - 2.29)	2.82 (2.80 - 2.85)
Avian influenza	4,57 (4.50 - 4.60)	1.69 (1.66 - 1.71)	2.59 (2.56 - 2.61)
Tuberculosis	4.49 (4.47 - 4.51)	1.68 (1.65 - 1.70)	2.50 (2.47 - 2.52)
Diabetes	4.18 (4.16 - 4.21)	2.11 (2.08 - 2.14)	2.85 (2.82 - 2.87)
High blood pressure	3.60 (3.57 - 3.62)	2.44 (2.41 - 2.47)	2.84 (2.82 - 2.87)
Food poisoning	3.59 (3.57 - 3.62)	2.38 (2.36 - 2.41)	2.73 (2.71 - 2.75)
Common cold	1.87 (1.85 - 1.89)	3.64 (3.61 - 3.67)	3.07 (3.05 - 3.09)

All four knowledge questions were answered correctly by 10.2% of the respondents; 16.5% had all answers wrong. The mean score for knowledge was 1.87 (95% CI 1.83 - 1.91, scale 0-4). Of all respondents 49% reported to have received a reasonable amount of information about AI and 7% (very) much, with a mean score for information received of 2.63 (95% CI 2.61 - 2.65, scale 1 - 5); 3% of respondents thought often or always about AI and 30% sometimes, resulting in a mean score of 2.19 (95% CI 2.17 - 2.22). 33% of respondents were not sure that they could do anything to prevent themselves from getting infected with AI, while 20% felt reasonably sure or very sure. The mean score for self efficacy was 2.23 (95% CI 2.20 - 2.27)

Almost half of the respondents (46%) reported taking one or more preventive measures, with 36% reporting to have stayed away from (wild) birds or poultry, 26% not going to areas where AI was present and 2% buying antiviral drugs (see Table 3). 54% of respondents did not take any measures, 13% took one measure, 14% took two measures, 10% took three and another 10% took four or more measures.

**Table 3:** Proportion of respondents that took preventive measures, overall and by measurement.

	*Mar '06*	*June '06*	Aug '06*	*Sept 06*	*Dec 06*	Feb 07*	*Mar 0*	*Overall*	*β-value*^*+*^	*Beta*^*§*^	*p-value*^*#*^
Took preventive measures	38%	50%	49%	43%	45%	48%	49%	46%	0.008	0.032	0.042
Not getting in touch with (wild) birds or poultry	31%	39%	39%	33%	34%	38%	38%	36%	0.005	0.022	0.175
Not going to areas with AI	18%	25%	28%	23%	27%	33%	31%	26%	0.018	0.081	0.000
Paying more attention to hygiene	9%	16%	17%	16%	14%	16%	18%	15%	0.008	0.042	0.003
Eating less or no chicken or poultry	9%	12%	11%	9%	14%	12%	12%	11%	0.004	0.022	0.175
Cancelled or didn't book a holiday to an area with AI	7%	9%	9%	6%	9%	11%	11%	9%	0.006	0.039	0.016
Getting oneself vaccinated against influenza	2%	6%	5%	6%	8%	4%	6%	5%	0.004	0.032	0.044
Keeping the cat indoors	5%	5%	4%	3%	4%	6%	6%	5%	0.001	0.013	0.428
Avoid gatherings of people	2%	2%	3%	3%	3%	3%	4%	3%	0.003	0.036	0.024
Buying antiviral drugs	1%	2%	2%	2%	2%	1%	4%	2%	0.002	0.034	0.036
Buying a mouth mask	1%	1%	2%	2%	2%	2%	3%	2%	0.003	0.045	0.005
Something else	2%	2%	2%	1%	3%	2%	2%	2%	0.000	0.001	0.975
Avoiding shaking hands	1%	1%	1%	1%	1%	1%	2%	1%	0.000	0.000	0.980

* additional surveys

^+ ^β-value: regression coefficient of time variable as a correlate of the independent variables, adjusted for gender, age and level of education

^§ ^Beta: standardised regression coefficient

^# ^p-value of the standardised regression coefficient from the linear regression analyses

**Figure 1:**
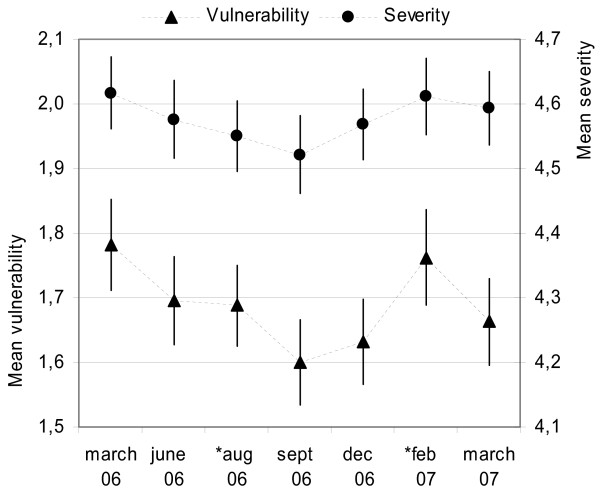
**Mean severity and vulnerability for AI with 95% confidence intervals**. * additional surveys

**Figure 2:**
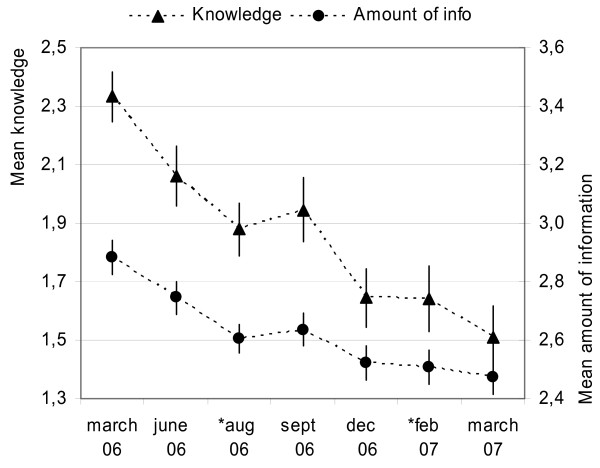
**Mean knowledge score and mean amount of information with 95% confidence levels**. * Additional surveys.

In the regression analyses, time was not significantly associated with perceived severity (β = -0.002, p = 0.772) and for perceived vulnerability the regression coefficient of time was just short of being statistically significant (β = -0.011, p = 0.08). Time was significantly associated with amount of information received (β = -0.065, p < 0.001), knowledge (β = -0.127, p < 0.001) and taking preventive measures (Table 3). In the pairwise comparisons, some more significant differences in variables of interest were found between different surveys. Perceived severity was stable over the seven surveys, ranging between 90% (August 2006) and 94% (March 2007). Perceived vulnerability decreased slightly between March 2006, when 2% perceived a (very) large chance of getting infected the coming year, and September and December 2006, with 0.4% and 0.4% respectively (ANOVA September and December 2006 vs. March 2006 p = 0.005 and p = 0.040 respectively). Perceived vulnerability was increased in February 2007 compared to September 2006 (ANOVA p = 0.04) (Figure 1). There was a significant decrease in the amount of information received about AI, from 2.88 in March 2006 to 2.47 in March 2007, in March 2006 14% had received (very) much information, in March 2007 this had decreased to 5% (Figure 2). Knowledge about AI also showed a significant decrease from 2.33 in March 2006 to 1.51 in March 2007, with in March 2006 43% of respondents answering three or four questions correct while in March 2007 this was 22%. The level of knowledge was positively associated with the amount of information received (Pearson r = 0.24, p < 0.001).

**Figure 3:**
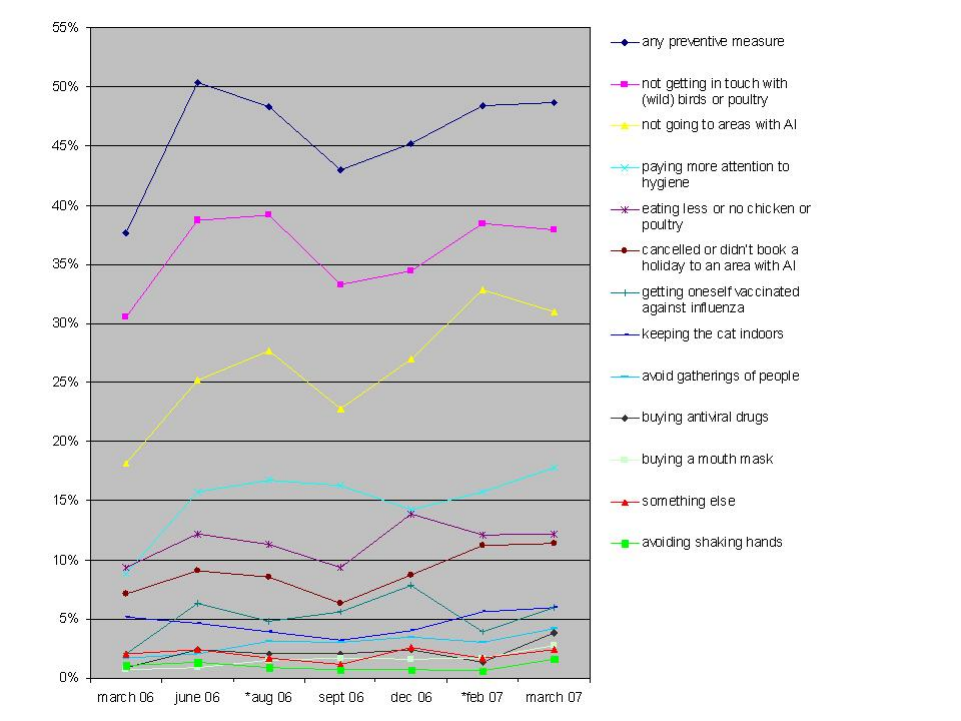
**Proportion of respondents that took preventive measures, overall and by measurement**. * Additional surveys

There was a significant increase in the percentage of respondents who had taken preventive measures between March and June 2006 from 38% to 50% (χ^2 ^(df) = 18.4 [1], p < 0.001), while there was no difference among the second until the last survey, ranging from 50% in the second survey to 43% in the fourth survey (χ^2 ^(df) = 8.2 [5], p = 0.147). Avoiding contact with (wild) birds or poultry was reported most often, by 36% of respondents (ranging between 33% in September 2006 and 39% in August 2006). For the specific preventive actions, an increase over time was observed for not going to areas with AI (March 2006 18%, February 2007 33%), paying more attention to hygiene (9% March 2006, 18% in March 2007), cancelled or did not book a holiday to an area with AI (6% in September 2006, 11% in March 2007), getting oneself vaccinated against influenza (2% March 2006, 8% December 2006), avoiding gatherings of people (2% March 2006, 4% March 2007), buying antiviral drugs (1% March 2006, 4% March 2007), and buying a mouth mask (1% March 2006, 3% March 2007) (Table 3; Figure 3). No differences in risk perceptions, precautionary actions or information received were observed related to the specific events (August 2006 and February 2007) when compared to the previous and consecutive surveys.

Most demographic factors and knowledge and information determinants were significantly associated with perceived vulnerability (Table 4). Perceived vulnerability was higher for women, for elder respondents, for respondents without children below 12, for those with a lower education, for those who thought more about AI, for those with a lower level of knowledge and for those vaccinated against influenza. Ethnicity and amount of information were not significantly associated with perceived vulnerability.

**Table 4:** Determinants of vulnerability for avian influenza.

	*mean*	*p-value*
Gender		0,019
Male	1,66	
Female	1,72	
Agegroup		0,002
18-29	1,66	
30-44	1,63	
45-59	1,73	
60 +	1,75	
Children <12 in household		0,024
Yes	1,64	
No	1,71	
Ethnicity		0,256
Dutch	1,68	
Non-Dutch	1,73	
Education*		<0.001
Low	1,84	
Intermediate	1,65	
High	1,62	
Think flu		<0.001
Never	1,46	
Rarely	1,62	
Sometimes	1,88	
Often/All the time	2,19	
Knowledge score		<0.001
0	1,80	
1	1,73	
2	1,72	
3	1,60	
4	1,53	
Amount of info		0,886
Nothing/Little	1,69	
Some	1,68	
(very) much	1,67	
Vaccinated against influenza		0,001
Yes	1,77	
No	1,67	

* Educational level is defined as: low: primary school, lower general secondary school, lower vocational school, or less; intermediate: high school or medium level vocational school; high: college or university degree.

In univariate logistic analysis of precautionary behaviour as the dependent (outcome) variable, the demographic variables, the specific surveys and variables from the Protection Motivation Theory were included as independent variables (Table 5). Of the demographic variables all, apart from gender and keeping chicken or poultry, were significantly associated with precautionary behaviour. Respondents with a higher age, a lower education, without children below 12, of non-Dutch ethnicity, and those who had been vaccinated against influenza were more likely to take preventive measures. Furthermore preventive measures were taken more often by those respondents who considered AI very severe, who had a higher perceived vulnerability, who had a higher self efficacy, who had less knowledge, who had received more information about AI and thought more about AI. Compared to the first survey, respondents in the subsequent surveys reported to have taken precautionary measures more often.

**Table 5:** Proportions of respondents that reported to have taken any preventive measures and results from logistics regressions analyses (Odds ratio (OR and 95% confidence intervals (95%CI)) exploring correlates of preventive measures.

				*univariate*				*multivariate*			
	n	N	%	OR	95%	CI	p-value	OR	95%	CI	p-value
Overall	1750	3811	45,9%								
Survey							<0,001				<0,001
1	218	579	37,7%	1,0				1,0			
2	272	540	50,4%	1,7	1,3	2,1		1,8	1,4	2,4	
3	314	644	48,8%	1,6	1,3	2,0		1,6	1,3	2,1	
4	230	535	43,0%	1,2	1,0	1,6		1,4	1,1	1,9	
5	248	549	45,2%	1,4	1,1	1,7		1,5	1,2	2,0	
6	224	463	48,4%	1,6	1,2	2,0		1,7	1,3	2,3	
7	244	501	48,7%	1,6	1,2	2,0		1,9	1,5	2,5	
Gender							0,493				
Male	821	1765	46,5%	1,0							
Female	929	2046	45,4%	1,0	0,8	1,1					
Age group							<0,001				<0,001
18-29	222	690	32,2%	1,0				1,0			
30-44	478	1178	40,6%	1,4	1,2	1,8		1,4	1,1	1,7	
45-59	581	1187	48,9%	2,0	1,7	2,5		1,8	1,5	2,2	
60 +	469	756	62,0%	3,4	2,8	4,3		2,9	2,2	3,7	
Education							<0,001				0,002
Low	538	1000	53,8%	1,0				1,0			
Intermediate	659	1479	44,6%	0,7	0,6	0,8		0,9	0,7	1,1	
High	553	1332	41,5%	0,6	0,5	0,7		0,7	0,6	0,9	
Children <12 in household							0,071				
No	1385	2966	46,7%	1,0							
Yes	365	845	43,2%	0,9	0,7	1,0					
Ethnicity							0,070				0,078
Dutch	1543	3398	45,4%	1,0				1,0			
Non-Dutch	207	413	50,1%	1,2	1,0	1,5		1,2	1,0	1,5	
Vaccinated against influenza							<0,001				0,010
Yes	506	887	57,0%	1,0				1,0			
No	1240	2909	42,6%	0,6	0,5	0,7		0,8	0,7	0,9	
Keeping chicken or poultry							0,715				
No	1685	3674	45,9%	1,0							
Yes	65	137	47,4%	1,1	0,8	1,5					
Severity							<0,001				<0,001
(Not) serious [1-4]	485	1256	38,6%	1,0				1,0			
Very serious [5]	1265	2555	49,5%	1,6	1,4	1,8		1,5	1,3	1,7	
Vulnerability							<0,001				<0,001
Very small [1]	839	1984	42,3%	1,0				1,0			
> Very small [2-5]	911	1827	49,9%	1,4	1,2	1,5		1,3	1,1	1,5	
Self efficacy							<0,001				
Not confident [1]	482	1273	37,9%	1,0							
> Not confident [2-5]	1268	2538	50,0%	1,6	1,4	1,9					
Self efficacy when never/rarely thinking of flu							<0,001				<0,001
Not confident [1]	251	815	30,8%	1,0				1,0			
> Not confident [2-5]	796	1738	45,8%	1,9	1,6	2,3		2,3	1,9	2,7	
Self efficacy when sometimes- all the time thinking of flu							<0,001				0,001
Not confident [1]	231	458	50,4%	1,0				1,0			
> Not confident [2-5]	472	800	59,0%	1,4	1,1	1,8		1,5	1,2	1,9	
Knowlegde score							<0,001				0,001
0-1	732	1496	48,9%	1,0				1,0			
2	514	1094	47,0%	0,9	0,8	1,1		0,9	0,8	1,1	
3-4	504	1221	41,3%	0,7	0,6	0,9		0,7	0,6	0,9	
Amount of info							<0,001				<0,001
Nothing/little [1,2]	695	1691	41,1%	1,0				1,0			
Some - very much [3-5]	1055	2120	49,8%	1,4	1,2	1,6		1,3	1,1	1,5	
Thinking of flu							<0,001				<0,001
Never/rarely [1,2]	1047	2553	41,0%	1,0				1,0			
Sometimes - all the time [2-5]	703	1258	55,9%	1,8	1,6	2,1		2,2	1,7	2,9	

The results of the multivariate logistic regression analysis are also shown in Table 5. As the odds ratio's of variables already in the model did not change substantially after inclusion of variables in subsequent steps we present the full model. The only statistically significant interaction term in the model was between self efficacy and thinking about AI. In the final model the time of the survey, a higher age, a lower level of education, a non-Dutch ethnicity, being vaccinated against influenza, a higher perceived severity, a higher perceived vulnerability, a higher self efficacy, a lower level of knowledge, more information about AI, and thinking more about AI were all associated with taking preventive measures. Self efficacy was a stronger predictor of precautionary behaviour for those who never or seldom think about AI (OR 2.3; 95% CI 1.9 - 2.7), compared to those who think about AI more often (OR 1.5; 95% CI 1.2 - 1.9). The discriminative value of the final model, expressed as the area under the curve (AUC) with 95% confidence limits is 0.69 (0.67-0.71).

## **Discussion**

The results of our study indicate that perceived severity of human infection with AI was and remained high; perceived vulnerability was low compared to diseases such as high blood pressure and a common cold. Perceived vulnerability decreased slightly during a one-year period covering part of 2006 and 2007. Comparative vulnerability was also relatively low indicating that people perceived it less likely that they would get infected with AI compared to others, which may be an indication of an optimistic bias. The amount of information received and the level of knowledge during the same period also decreased. Substantial groups reported taking one or more preventive measures with staying away from wild birds and poultry remaining high throughout the period. Our results further indicate that older people, women, people without younger children, those with a lower education, who thought more about AI, with a lower level of knowledge about AI, and who were vaccinated against influenza perceived their vulnerability for AI as higher. Furthermore, respondents who were older, lower educated, of non-Dutch ethnicity, vaccinated against influenza, had higher risk perceptions or self efficacy, less knowledge, had received more information about AI, and thought more often about AI, were more likely to report engagement in precautionary actions.

A number of Asian studies looked into risk perception of SARS over time. Lau and colleagues studied risk perception of SARS in Hong Kong during the outbreak with ten rounds of surveys and showed changes in both risk perception and precautionary behaviour [34]. They showed that perceived susceptibility declined in the second phase of the epidemic, after April 8, 2003 as the number of new infections also declined. During the initial phase of the epidemic with rising figures of new cases there was a sharp increase in preventive measures. Engagement in preventive measures remained on a high level, also after the epidemic started declining and perceived susceptibility also declined. Leung and colleagues also studied SARS in Hong Kong with six surveys and showed a decrease in anxiety over time after the peak of the SARS epidemic [36]. They also showed an increase in the number of preventive measures at the start of the epidemic, which remained stable during the epidemic, and this decreased sharply six months after the epidemic.

A number of studies have explored risk perception of AI or an influenza pandemic and future preventive behaviours. A Norwegian study showed that most people regarded an influenza pandemic as a serious health issue, although almost half of them underestimated the expected mortality compared to the official estimations [37]. 80% of respondents reported that they would be careful about personal hygiene, while 11% reported to stay home and avoid contact with others. Gupta and colleagues in a street-based survey in London concluded that 71% of their respondents thought an influenza pandemic (very) likely in the coming ten years, and almost all respondents reported that they would wash their hands more than five times per day if requested [38]. Fielding studied risk perception of AI in relation to live chicken sales and reported that 36% considered touching chicken while buying them as risky [39].

Lau and colleagues carried out three studies in Hong Kong on different aspects of human AI and human-to-human transmission of AI [31,40,41]. In a first study substantial unconfirmed beliefs and misconceptions were reported related to AI which were correlated to immediate behavioural responses, such as avoiding visits to hospitals and eating less poultry [40]. A second study indicated that if human-to-human transmission would occur in Hong Kong, large proportions of respondents would wear face masks in public venues (74%), increase the frequency of hand washing (87%) or avoid eating poultry (64%) [31]. The results of the third study showed that between 71 and 81% of respondents reported to avoid visiting hospitals, crowds, going out or going abroad when there was either bird-to-human transmission or human-to-human transmission [41].

The high level of perceived severity of AI in our study is in line with earlier studies that were conducted in Norway, the UK and Hong Kong [31,37,38]. In our own earlier international comparative study perceived severity for AI in the Netherlands was also high [42]. As in other studies women, the elderly and those with a lower education have a higher perceived vulnerability [19,43]. Many of the determinants we identified in this study are in line with the studies of Lau and colleagues conducted in Hong Kong although they focused on human-to-human transmission of AI [31,41]. Of the common demographic variables (gender and age), in these studies older respondents also reported higher intentions to take preventive measures. In their studies anticipated preventive behaviour when human-to-human transmission would occur was also related to a higher perceived susceptibility to H5N1 infection for oneself or one's family, which is in line with our findings on perceived vulnerability.

While perceived severity remained stable and perceived vulnerability decreased slightly, there was a stronger decrease in the amount of information received and related decrease in knowledge. The level of knowledge was positively associated with the amount of information received (Pearson r = 0.24, p < 0.001). This relation may be associated with the decrease in media attention to avian influenza during this period. An inventory of attention for AI and an influenza pandemic in two of the main national newspapers, Algemeen Dagblad and NRC Handelsblad showed this decrease. While in 2006 from March until December these newspapers published 150 and 107 articles respectively, this number decreased in 2007 to 74 and 55 respectively, and to 8 and 6 in the first three months of 2008.

There was, however, no significant effect on either perceived severity, perceived vulnerability, nor on the amount of information or knowledge of the two episodes: the suspicion of AI among owls in the Rotterdam zoo and the outbreak among poultry in the UK leading to new requirements to keep birds under cover. During the SARS outbreak in Hong Kong changes in risk perception where shown to be related to the course of the outbreak [34,36], whereas even the introduction of AI in Europe did not appear to lead to a change in risk perception [42]. This might indicate that only a true outbreak of an emerging infectious disease and changes in its course will lead to changes in risk perception.

The stable high level of preventive measures is in line with the earlier studies on SARS in Hong Kong where these measures remained high during the epidemic and, as Leung showed, only decreased after the epidemic ended [36]. More research, however, is needed to establish in more detail how changes in the course of outbreaks in and outside the country and media coverage are related to risk perceptions and preventive behaviour.

Our study makes clear that AI is seen as a serious but rare disease, similar to such infectious diseases as tuberculosis and HIV. These diseases are rare among the general population in the Netherlands, leading to high perceived severity but lower perceived vulnerability [44,45]. HIV and tuberculosis can be considered diseases with high prevalence in specific population groups, leading to low comparative vulnerability in the general population: for the population at large such diseases, especially HIV, are perceived as a threat to 'others'. A similar low comparative vulnerability as for tuberculosis was observed for AI.

An important result of our study is the difference between thinking about AI and knowledge about AI in relation to perceived vulnerability. While more 'actual' knowledge about the disease is associated with lower perceived vulnerability, thinking more often about AI correlates with a higher perceived vulnerability. 'Thinking about' AI may be a proxy for worry about the disease. Worry can be related to both risk perception and preventive behaviours [25,46]. Lau and colleagues also found that worry about oneself or family members contracting the virus was associated with anticipated preventive behaviour [31]. The interaction effect between thinking about AI and self efficacy also underlines that thinking about AI, or worry, warrants separate attention.

The present study is unique in that we included self-reported preventive behaviours related to AI rather than only intentions or plans. Our findings confirm that next to demographic variables perceived severity, perceived vulnerability, amount of information, thinking about AI and self efficacy are significantly associated with precautionary behaviour. The fact that less knowledge was related to taking preventive measures more often asks for more detailed research into the relation between knowledge and preventive behaviours. It might be that in this case - because there were no cases of avian influenza in the Netherlands - that those with more specific knowledge considered preventive measures less necessary.

If additional research suggests that these relations are causal, it may inform public health interventions, because these determinants can be influenced by communication strategies, which is of great importance for non-pharmaceutical intervention strategies during outbreaks or a pandemic. Evidently, risk perceptions in terms of probability and severity is only one of many potential determinants of precautionary behaviours. A realistic perception of risk is, however, recognised in key behaviour change theories as a crucial step towards protection motivation [15,47,48]. The moderate discriminative value (AUC = 0.69) of the multivariate model describing factors associated with precautionary behaviour however suggests that other, unmeasured factors may be of additional importance.

Our study has several limitations. Firstly we used an Internet based panel, which may have led to an overrepresentation of those who are computer literate. The panel, however, is representative for the Dutch population in terms of age, gender and level of education. The percentage of people being vaccinated against influenza in our study (23%) is somewhat higher than what has been reported for the general Dutch population (18,1%) [45] The fact that we used a research panel of which participants in the present study received a very small reimbursement for completing surveys (i.e. Euro 1,50 per survey), may have resulted in a study population that is somewhat more interested in participating in research surveys. However, using such a panel for this series of surveys has also most probably resulted in higher response rates than if we used sample from the general population.

A second limitation is that in our question about precautionary behaviour we did not specify when the respondents engaged in such behaviour. This may have resulted in over-reporting of preventive measures especially in the latter surveys. A third limitation was that we considered those answers to the knowledge questions right which were in line with the general public information, however, during the times of the surveys there was some scientific evidence about isolated non-sustained human-to-human transmission. This might have led to some people answering this question differently. Finally, we used a questionnaire that has not been validated in other studies, although it has been based on earlier studies related to risk perception of SARS, AI and other (infectious) diseases [33,42].

## Conclusions

Our study has several implications for public health policy and research. The results of this study support the validity of the Protection Motivation Theory for investigations of potential determinants of precautionary behaviours for emerging infectious diseases. The fact that perceived severity of AI appears to be high offers a good point of departure for more specific risk communications to promote precautionary actions if needed. The stability of the level of risk perception indicates that it is not seen as a temporary problem. Knowledge however, decreased over time indicating the need to keep the public continually well informed, especially about which measures will be effective, since a substantial number of respondents took non-effective measures. In the current H1N1 pandemic this underlines the necessity to inform the general public about the specific features of the pandemic as well as preventive measures. The stability of the levels of risk perception gives credibility to cross-sectional one-time surveys of risk perception.

## Competing interests

The authors declare that they have no competing interests.

## Authors' contributions

OdZ conceived and designed the study and drafted the manuscript. IV participated in the design of the study, coordinated the surveys and performed the statistical analysis. JHR helped to draft the manuscript. JB helped in the conception of the study, participated in its design and helped to draft the manuscript. All authors read and approved the final manuscript.

## Pre-publication history

The pre-publication history for this paper can be accessed here:

http://www.biomedcentral.com/1471-2334/10/114/prepub
